# Efficacy of Voclosporin in Proliferative Lupus Nephritis with High Levels of Proteinuria

**DOI:** 10.2215/CJN.0000000000000297

**Published:** 2023-12-18

**Authors:** Hanni Menn-Josephy, Lucy S. Hodge, Vanessa Birardi, Henry Leher

**Affiliations:** 1 Boston University School of Medicine, Boston, Massachusetts; 2 Aurinia Pharmaceuticals Inc., Edmonton, Alberta, Canada

**Keywords:** lupus nephritis, renal failure, renal function, renal function decline, systemic lupus erythematosus

## Abstract

**Background:**

In a phase 3 study of adults with active lupus nephritis, addition of voclosporin to mycophenolate mofetil (MMF) and low-dose glucocorticoids led to significant improvements in the proportion of participants achieving complete and partial renal response as well as sustained reduction in proteinuria. This analysis examined the efficacy and safety of voclosporin in a subgroup of the phase 3 study with proliferative lupus nephritis and high levels of proteinuria.

**Methods:**

Participants were randomized to oral voclosporin (23.7 mg twice daily) or placebo for 12 months; all participants received MMF and low-dose glucocorticoids. This analysis includes participants with class III or IV (±class V) lupus nephritis and baseline urine protein–creatinine ratio (UPCR) ≥3 g/g. Efficacy end points included complete renal response (UPCR ≤0.5 g/g with stable eGFR, low-dose glucocorticoids, and no rescue medication), partial renal response (≥50% reduction from baseline UPCR), and UPCR over time. Safety outcomes were also assessed.

**Results:**

A total of 148 participants were in the voclosporin (*n*=76) and control (*n*=72) arms. At 12 months, 34% and 11% of participants in the voclosporin and control arms, respectively, achieved a complete renal response (odds ratio, 4.43; 95% confidence interval [CI], 1.78 to >9.99; *P* = 0.001). A partial renal response was achieved by 65% of the voclosporin arm and 51% of the control arm at 12 months (odds ratio, 1.60; 95% CI, 0.8 to 3.20; *P* = 0.18). More voclosporin- than control-treated participants achieved UPCR ≤0.5 g/g (51% versus 26%), and voclosporin-treated participants met this end point significantly earlier (hazard ratio, 2.07; 95% CI, 1.19 to 3.60; *P* = 0.01). The incidence of adverse events was similar between the arms; mean eGFR values remained stable and within normal range in both arms.

**Conclusions:**

Addition of voclosporin to MMF and low-dose glucocorticoids resulted in a significantly higher proportion of participants with proliferative lupus nephritis achieving complete and partial renal responses as well as earlier reductions in proteinuria, with no evidence of worsening kidney function.

## Visual Abstract

**Figure:**
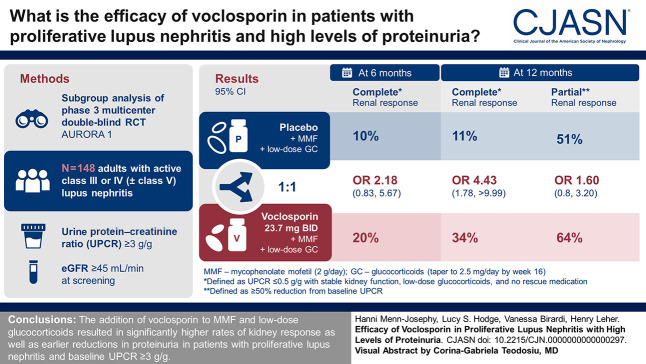

## Introduction

Lupus nephritis is a severe, yet common, renal complication of systemic lupus erythematosus. Approximately 45% of patients with systemic lupus erythematosus will experience lupus nephritis throughout their disease course, with 10%–30% of patients with lupus nephritis progressing to kidney failure within 15 years of diagnosis.^1–5^

In proliferative lupus nephritis, ongoing immunologic insult to the kidney leads to inflammation and fibrosis, ultimately resulting in proteinuria, one of the most common clinical manifestations of the disease.^6,7^ Proteinuria has been identified as an independent mediator of progressive kidney damage in lupus nephritis.^8–10^ Nephrotic-range proteinuria, typically defined as urine protein–creatinine ratio (UPCR) ≥3 g/g, has been associated with a lower likelihood of achieving complete or partial renal response in patients with lupus nephritis and an overall worse prognosis.^4,8–16^ Furthermore, in lupus nephritis and in other inflammatory diseases leading to CKD, higher levels of proteinuria have been associated with significantly greater and more rapid decline in eGFR over time.^4,17–20^

Proteinuria remains one of the most commonly used surrogate markers of disease activity in lupus nephritis, and early reductions in proteinuria after treatment initiation have been shown to predict improved long-term renal outcomes.^13,21–25^ The current European League Against Rheumatism and the Kidney Disease Improving Global Outcomes Clinical Practice Guideline for the management of lupus nephritis are focused on lowering proteinuria, with the European League Against Rheumatism recommending a goal of ≥50% reduction in UPCR by 6 months and 24-hour protein of <0.5–0.7 g with near-normal eGFR by 12 months.^15,26^

Voclosporin is a second-generation calcineurin inhibitor approved in the United States, Great Britain, the European Union, and Switzerland for the treatment of adult patients with active lupus nephritis in combination with background immunosuppressive therapy. Voclosporin was developed through the addition of a single carbon extension to the amino acid-1 position of cyclosporin A. This modification led to a more consistent pharmacokinetic/pharmacodynamic relationship, alleviating the need for therapeutic drug monitoring required of other calcineurin inhibitors, as well as to a better tolerability profile at doses used to treat lupus nephritis.^27^

Positive results from the phase 2 Aurinia Urinary Protein Reduction in Active Lupus with Voclosporin (AURA-LV) trial led to the pivotal phase 3 study (Aurinia Renal Response in Active Lupus with Voclosporin [AURORA 1]), a global, 52-week, double-blind, randomized controlled clinical trial, which further assessed the efficacy and safety of voclosporin 23.7 mg twice daily compared with placebo in achieving complete renal response in active lupus nephritis when used with mycophenolate mofetil (MMF) and rapidly tapered oral glucocorticoids.^28,29^ Significantly more voclosporin-treated participants achieved a complete renal response at 52 weeks (odds ratio [OR], 2.65; 95% confidence interval [CI], 1.64 to 4.27; *P* < 0.0001). Furthermore, participants treated with voclosporin achieved reductions in proteinuria significantly earlier. Additional subanalyses across biopsy subclasses, races, and ethnicities demonstrated similar efficacy of voclosporin.^30^

Given the significance of early reductions in proteinuria on long-term renal outcomes in lupus nephritis, we have performed a *post hoc* analysis of the AURORA 1 trial to investigate the efficacy and safety of voclosporin in patients with proliferative and baseline proteinuria ≥3 g/g.

## Methods

### Study Design

This study is a *post hoc* analysis of AURORA 1, a phase 3, randomized, prospective, placebo-controlled, double-blind, multicenter, international, two-arm comparison trial (ClinicalTrials.gov and EudraCT identifiers: NCT03021499 and 2016-004045-81, respectively) conducted between May 2017 and October 2019.

Study design and methods have previously been described.^29^ In brief, participants were randomly assigned (1:1) to either oral voclosporin 23.7 mg twice daily or to matching placebo; all participants received MMF (target dose of 2 g/d) and oral glucocorticoids (Supplemental Figure 1). Glucocorticoids were administered according to a protocol-defined tapering schedule (intravenous methylprednisolone on days 1 and 2, then oral prednisone on day 3, starting at 20–25 mg/d and decreasing to 2.5 mg/d by week 16; Supplemental Figure 2). Owing to the expected hemodynamic effects of calcineurin inhibitors on eGFR, the protocol provided guidance for study drug dose modification for participants experiencing decreases in eGFR or increases in BP.^31^

As part of the AURORA 1 study implementation, an institutional review board or independent ethics committee at each participating site approved the informed consent form and protocol. All participants provided written informed consent in accordance with the Declaration of Helsinki.^29^

### Participants

Participants enrolled in the AURORA 1 trial were 18 years and older, diagnosed with active lupus nephritis with a kidney biopsy within the previous 2 years demonstrating class III, IV, or V (alone or in combination with class III or IV) lupus nephritis, had proteinuria ≥1.5 g/g (≥2 g/g for class V) by first morning void, and had eGFR ≥45 ml/min per 1.73 m^2^ at screening; biopsy class was determined by the local pathologist at each site.

Participants included in this *post hoc* analysis had baseline UPCR ≥3 g/g with active, biopsy-proven class III or IV lupus nephritis (±class V lesions). Given that pure class V (non-proliferative) lesions have distinct clinical trajectories and prognosis, we excluded these participants from this analysis. For comparison, additional analyses, including participants with UPCR <3 g/g, are included in the supplement.

### End Points

Efficacy end points included complete renal response (defined as a composite end point of UPCR ≤0.5 g/g with eGFR ≥60 ml/min per 1.73 m^2^ or no decrease >20% from baseline, use of low-dose glucocorticoids [defined as no more than 10 mg prednisone equivalent per day for ≥3 consecutive days or for ≥7 days in the 8 weeks before the end point assessment], and no use of rescue medication) and partial renal response (defined as ≥50% reduction in UPCR from baseline). In addition, time to ≥50% reduction in UPCR from baseline, time to UPCR ≤0.5 g/g, and mean UPCR over time were analyzed. A masked, independent Clinical Endpoints Committee adjudicated the end point of complete renal response at 6 and 12 months, on the basis of an assessment of UPCR, eGFR, steroid dose, rescue and prohibited medications, and adverse events for each participant. Changes from baseline in UPCR and changes from screening visit in immunology parameters (complement 3, complement 4, and anti–double-stranded deoxyribonucleic acid) were also assessed.

Safety end points included adverse events, reported at the discretion of the study investigator using preferred terms aggregated by System Organ Class in the Medical Dictionary for Regulatory Activities version 20.0 and biochemical and hematologic laboratory assessments. Adverse events by preferred term of GFR decreased were reductions in eGFR determined to be clinically relevant at the discretion of the study investigator and were not based on a specific magnitude of reduction from baseline. Laboratory-confirmed decreases in eGFR of ≥30% from baseline were determined programmatically on the basis of reported laboratory values from two study visits.

Changes from baseline in the laboratory assessments and vital signs reported in this article were assessed at the following time points: baseline and weeks 2, 4, 8, 12, 16, 20, 24, 30, 36, 42, 48, and 52.

### Statistical Analyses

The proportion of patients achieving partial and complete renal response were calculated using a logistic regression model with terms for treatment arm (voclosporin or control), covariates, and treatment-by-covariate interaction. Participants withdrawing for any reason or those without data at month 6 or 12 were counted as nonresponders at those time points. Change from baseline measures were calculated using a mixed-effect model repeated-measures analysis, including terms for treatment, covariates of interest, visit, and treatment by interaction. Median time-to-event measures (95% CI) were calculated using the Kaplan–Meier method; hazard ratios (HRs) and 95% CIs were derived from Cox proportional hazards model with terms for treatment, baseline UPCR, biopsy class, MMF use at screening, and region. Descriptive statistics, including means, SDs, counts, and percentages, were used to describe various aspects of the populations of interest.

To account for baseline hyperfiltration known to precede the onset of CKD, analyses of corrected eGFR (using the Chronic Kidney Disease Epidemiology Collaboration equation) constrained all values >90 ml/min per 1.73 m^2^ to a maximum of 90 ml/min per 1.73 m^2^ to mitigate the risk of false negativity.

## Results

**Figure 1:**
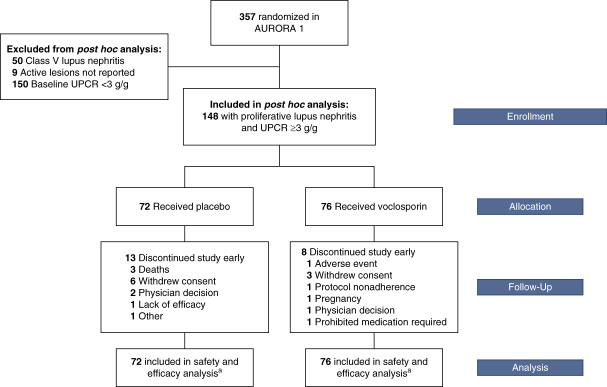
**Disposition of participants with proliferative lupus nephritis and UPCR ≥3 g/g.** ^a^All 148 participants who met the inclusion and exclusion criteria were included in the efficacy and safety analyses unless otherwise specified. AURORA 1, Aurinia Renal Response in Active Lupus with Voclosporin; UPCR, urine protein–creatinine ratio.

A total of 148 participants (voclosporin, *n*=76; control, *n*=72) of the AURORA 1 study were included in this analysis (Figure 1). Demographic characteristics were similar in the voclosporin and control arms (Table 1). Most of the participants had a histopathologic diagnosis of class IV lupus nephritis with or without class V lesions. Mean baseline UPCR levels (voclosporin, 6.2 g/g versus control, 5.7 g/g) and mean time since diagnosis of lupus nephritis (3.0 and 3.3 years, respectively) were similar between the arms. Most of the participants in both arms had complement 3 levels <90 mg/dl (63% and 67%, respectively) and anti–double-stranded deoxyribonucleic acid ≥10 IU/ml (84% and 68%, respectively). Overall, 25% of participants had a baseline eGFR <60 ml/min per 1.73 m^2^. Hydroxychloroquine and angiotensin-converting enzyme inhibitor/angiotensin receptor blocker use at baseline was similar. Compared with participants from the AURORA 1 study with baseline UPCR <3 g/g (Supplemental Table 1), participants with proliferative disease and UPCR ≥3 g/g were more likely to have class IV disease and to have self-identified as non-White (Table 1).

**Table 1:** Baseline demographics and disease characteristics of participants with proliferative lupus nephritis and urine protein–creatinine ratio ≥3 g/g

Characteristic	Control *n*=72	Voclosporin *n*=76	Overall *n*=148
Age, mean (SD) yr	31 (10)	31 (10)	31 (10)
Female sex, *n* (%)	62 (86)	67 (88)	129 (87)
**Race, *n* (%)**			
Asian^a^	28 (39)	20 (26)	48 (32)
Black	7 (10)	11 (15)	18 (12)
Other^b^	17 (24)	16 (21)	33 (22)
White	20 (28)	29 (38)	49 (33)
**Ethnicity, *n* (%)**			
Hispanic or Latino	21 (29)	23 (30)	44 (30)
Not Hispanic or Latino	51 (71)	53 (70)	104 (70)
Time since lupus nephritis diagnosis, mean (SD) yr	3.3 (4.5)	3.0 (3.7)	3.2 (4.1)
**Biopsy class, *n* (%)**			
Class III	13 (18)	6 (8)	19 (13)
Class IV	39 (54)	50 (66)	89 (60)
Class III/V	8 (11)	11 (14)	19 (13)
Class IV/V	12 (17)	9 (12)	21 (14)
UPCR, mean (SD) g/g	5.7 (2.3)	6.2 (2.6)	5.9 (2.4)
**eGFR**			
Mean (SD) ml/min per 1.73 m^2^	91 (29)	84 (30)	88 (30)
<60 ml/min per 1.73 m^2^, *n* (%)	15 (21)	22 (29)	37 (25)
Serum creatinine, mean (SD) mg/dl	0.9 (0.4)	1.0 (0.3)	0.9 (0.3)
**Complement 3**			
Mean (SD) mg/dl	79 (36)	77 (32)	78 (34)
Low <90 mg/dl, *n* (%)	48 (67)	48 (63)	96 (65)
**Complement 4**			
Mean (SD) mg/dl	16 (11)	17 (9)	17 (10)
Low <10 mg/dl, *n* (%)	22 (31)	19 (25)	41 (28)
**Anti-dsDNA**			
Mean (SD) IU/ml	107 (134)	115 (126)	111 (129)
High ≥10 IU/dl, *n* (%)	49 (68)	64 (84)	113 (76)
**ACEi/ARB use**			
Yes	46 (64)	49 (65)	95 (64)
No	26 (36)	27 (36)	53 (36)
**Hydroxychloroquine use**			
Yes	41 (57)	41 (54)	82 (55)
No	31 (43)	35 (46)	66 (45)

ACEi/ARB, angiotensin-converting enzyme inhibitor/angiotensin II receptor blocker; anti-ds DNA, anti–double-stranded deoxyribonucleic acid; UPCR, urine protein–creatinine ratio.

a Asian race includes Asian Indian, Chinese, Filipino, Japanese, Korean, Vietnamese, and other Asian.

b Other race includes American Indian, Alaska Native, Native Hawaiian, Pacific Islander, and other or mixed races, except mixed Black race.

### Exposure

Overall, 127 participants (86%) completed the study (90% and 82% of the voclosporin and control arms, respectively), with 71% and 61% of each arm, respectively, completing 1 year of study drug treatment (Supplemental Table 2). The mean (SD) duration of exposure to the study drug was similar for both voclosporin- and control-treated participants (317 [98] versus 294 [111] days, respectively). Exposure to MMF was comparable, with a mean (SD) final dose of 1.84 g/d (0.54) in the voclosporin arm and 1.92 g/d (0.56) in the control arm.

Similar proportions of participants in the voclosporin and control arms were on oral prednisone (or equivalent) doses of ≤2.5 mg/d at 6 months (87% and 86%, respectively) and 12 months (66% and 70%, respectively; Supplemental Table 3). The mean (SD) duration of exposure to oral glucocorticoids in the voclosporin and control arms was 342 days (86) and 329 days (98), respectively.

### Renal Response End Points

**Figure 2:**
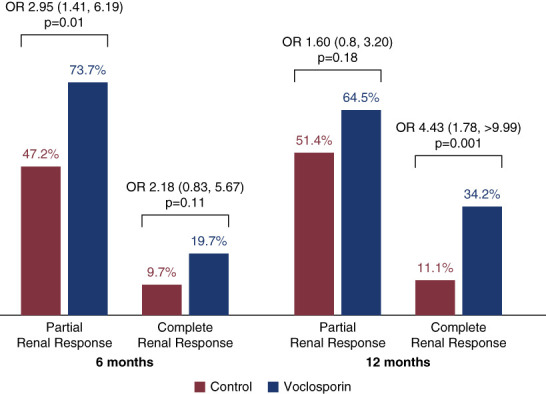
**Complete renal response and partial renal response at 6 and 12 months in participants with proliferative lupus nephritis and UPCR ≥3 g/g.** Complete renal response defined as UPCR ≤0.5 g/g with stable renal function (eGFR ≥60 ml/min per 1.73 m^2^ or no decrease >20% from baseline) in the presence of sustained, low-dose glucocorticoids (in the 8 weeks before assessment) and no rescue medication. Partial renal response defined as a ≥50% reduction in UPCR from baseline. Analysis was conducted using a logistic regression model with terms for treatment, baseline UPCR, biopsy class, MMF use at baseline, and region. Statistical data presented as OR (95% confidence interval). ORs >1 demonstrate a treatment benefit of voclosporin. MMF, mycophenolate mofetil; OR, odds ratio.

Compared with the control arm (10%), a greater proportion of patients treated with voclosporin achieved a complete renal response at 6 months (20%, OR, 2.18; 95% CI, 0.83 to 5.67; *P* = 0.11). At 12 months, 34% of patients in the voclosporin arm had achieved a complete renal response compared with 11% in the control arm (OR, 4.43; 95% CI, 1.78 to >9.99; *P* = 0.001, Figure 2). Similarly, higher proportions of voclosporin-treated participants achieved a partial renal response at 6 and 12 months compared with the control arm (Figure 2). There was also a treatment benefit with voclosporin observed in patients in the AURORA 1 study with UPCR <3 g/g, with 49% of voclosporin-treated patients achieving a complete renal response at 12 months compared with 33% of control-treated participants (OR, 1.98; 95% CI, 1.06 to 3.68; *P* = 0.03; Supplemental Table 4).

**Figure 3:**
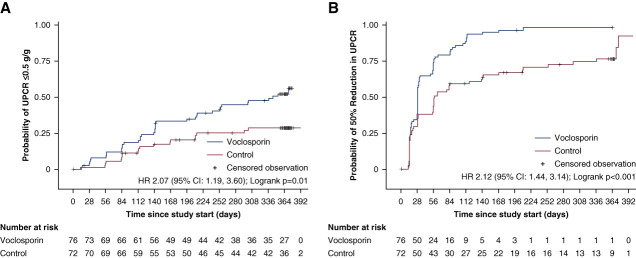
**Probability of achieving UPCR reductions in participants with proliferative lupus nephritis and UPCR ≥3 g/g**. Probability of (A) UPCR ≤0.5 g/g and (B) ≥50% reduction in UPCR from baseline in participants with proliferative lupus nephritis and UPCR ≥3 g/g. Median time to event (95% CI) calculated using Kaplan–Meier methods. Participants who did not achieve the event were censored on the day of their last available UPCR assessment. Hazard ratios and 95% CIs were derived from a Cox proportional hazards model with terms for treatment group, baseline UPCR, biopsy class, mycophenolate mofetil use at screening, and region. CI, confidence interval; HR, hazard ratio.

Overall, UPCR ≤0.5 g/g was achieved in 51% of participants in the voclosporin arm and 26% of participants in the control arm. The median time to UPCR ≤0.5 g/g for participants receiving voclosporin was 344 days (11.3 months); in the control arm, a median time to achieving UPCR ≤0.5 g/g could not be determined because <50% of participants achieved the end point within the study period (HR, 2.07; 95% CI, 1.19 to 3.60; *P* = 0.01, Figure 3A, Table 2).

**Table 2:** Time to UPCR reductions in participants with proliferative lupus nephritis and UPCR ≥3 g/g

UPCR Reduction Measure	Control *n*=72	Voclosporin *n*=76	Hazard Ratio versus Control (95% CI)
**UPCR ≤0.5 g/g**			
Participants with UPCR ≤0.5 g/g, *n* (%)	19 (26)	39 (51)	
Median (95% CI) time to UPCR ≤0.5 g/g, d	ND	344 (214 to ND)	2.07 (1.19 to 3.60)
**UPCR reduction ≥50% from baseline**			
Participants with UPCR reduction ≥50%, *n* (%)	54 (75)	74 (97)	
Median (95% CI) time to ≥50% reduction in UPCR, d	58 (30 to 139)	29 (28 to 32)	2.12 (1.44 to 3.14)

Median time to event (95% CI) calculated using Kaplan–Meier methods. Participants who did not achieve the event during the 12-month study period were censored on the day of their last available UPCR assessment. Hazard ratios and 95% CIs were calculated to evaluate the significance of the difference in median times to event and were derived from a Cox proportional hazards model with terms for treatment group, baseline UPCR, biopsy class, mycophenolate mofetil use at screening, and region. CI, confidence interval; ND, not determinable due to either the end point not being met by 50% of the cohort within the study period or limited additional participants achieving the end point after the median time point but before the end of the study; UPCR, urine protein–creatinine ratio.

**Figure 4:**
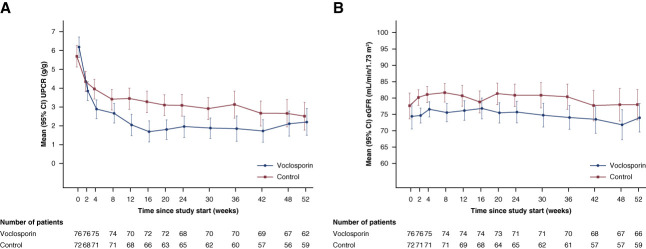
**UPCR and eGFR over time in participants with proliferative lupus nephritis and UPCR ≥3 g/g.** Least squares mean of (A) UPCR and (B) corrected eGFR in participants with proliferative lupus nephritis and UPCR ≥3 g/g. Only participants with available data at the specified time points are included in the analyses. Baseline least squares means and 95% CIs are calculated from a model including a covariate for treatment group. Post-treatment least squares means and 95% CIs are calculated from a model including covariates for treatment group and baseline value. Renal function assessed with corrected eGFR (Chronic Kidney Disease Epidemiology Collaboration equation) using a prespecified ceiling of 90 ml/min per 1.73 m^2^.

A ≥50% reduction in UPCR from baseline at any time during the study period was achieved in 97% and 75% of participants in the voclosporin and control arms, with median times to this end point of 29 and 58 days, respectively (HR, 2.12; 95% CI, 1.44 to 3.14; *P* < 0.001, Figure 3B, Table 2). Mean UPCR levels decreased over time in both arms with lower mean values observed in the voclosporin arm at all time points after treatment initiation (Figure 4A).

Similar improvements in immunology parameters were observed in both arms (Supplemental Table 5).

### Safety

Overall, the incidence of adverse events was comparable between arms, with 96% of the voclosporin arm and 92% of the control arm experiencing an adverse event during the study period (Table 3). The incidence of adverse events leading to study drug discontinuation was similar between the arms (voclosporin, 15% versus control, 21%; Supplemental Table 7). Serious adverse events were observed in 18% of the voclosporin arm and 24% of the control arm. The incidence of adverse events associated with voclosporin treatment was similar in participants in the AURORA 1 study with UPCR ≥3 g/g and those with UPCR <3 g/g (Supplemental Table 8).

**Table 3:** Adverse events in participants with proliferative lupus nephritis and UPCR ≥3 g/g

Adverse Events	Control *n*=72, *n* (%)	Voclosporin *n*=76, *n* (%)
Adverse event (AE)	66 (92)	73 (96)
Serious adverse event (SAE)	17 (24)	14 (18)
Treatment-related SAE	4 (6)	2 (3)
AE leading to study drug discontinuation	15 (21)	11 (15)
Death	3 (4)	0
Treatment-related AE leading to death	0	0

Adverse events are defined as an adverse event that occurs on or after the day of the first dose and up to 30 days after the last dose of study drug, with the exception of death; it includes all deaths after randomization until completion of study follow-up. UPCR, urine protein–creatinine ratio.

Adverse events in the Medical Dictionary for Regulatory Activities System Order Class of Infections and Infestations were most common, reported by 62% in the voclosporin arm and 58% in the control arm (Supplemental Table 6), and the most commonly reported infection by preferred term was upper respiratory tract infection (voclosporin, 17% versus control, 19%). Serious infections were reported by five (7%) and eight (11%) participants in the voclosporin and control arms, respectively.

No deaths were reported in the voclosporin arm; three deaths, attributed to acute respiratory failure, pulmonary embolism, and pneumonia/septic shock, occurred in the control arm.

Mean corrected eGFR values remained stable and within the normal range at all time points in both arms (Figure 4B). Adverse events of GFR decreased (preferred term reported per investigator discretion) occurred in 32% of participants in the voclosporin arm compared with 6% of participants in the control arm. Serious adverse events of GFR decreased were reported in one patient in each arm. Laboratory-confirmed decreases in eGFR of ≥30% from baseline were reported in 16% and 18% of the voclosporin and control arms, respectively.

## Discussion

This *post hoc* analysis of the phase 3 AURORA 1 clinical trial evaluated the efficacy and safety of voclosporin in a subgroup of 148 participants with proliferative lupus nephritis and high levels of proteinuria. In this study, participants treated with voclosporin achieved significantly higher rates of complete renal response at 12 months than participants treated with placebo when administered in combination with MMF and low-dose glucocorticoids. In addition, treatment with voclosporin was associated with earlier and greater reductions in proteinuria that were maintained throughout the study.

Current guidelines for the management of lupus nephritis recommend targeting a UPCR of <0.5–0.7 g/g within 12 months of initiating treatment.^15,26^ In this study, 34% of participants in the voclosporin arm achieved a complete renal response (requiring UPCR reduction to <0.5 g/g) at 12 months compared with 11% in the control arm. While the rates of complete renal response at 12 months were lower in participants with UPCR ≥3 g/g than in the overall AURORA 1 population (voclosporin, 41% versus control, 22%), the difference in the percentage of participants with UPCR ≥3 g/g meeting this end point was associated with a higher OR (4.43; 95% CI, 1.78 to >9.99; *P* = 0.001 versus 2.65; 95% CI, 1.64 to 4.27; *P* < 0.0001 in AURORA 1).^29^ Furthermore, these complete renal response rates were obtained using a novel glucocorticoid tapering regimen where participants may have received IV glucocorticoids on days 1 and 2 then initiated oral glucocorticoids at a dose of only 20–25 mg/d on day 3 and rapidly tapered to 2.5 mg/d by week 16 of treatment. These results support the growing body of evidence consistent with current guidelines that lower doses of glucocorticoids are efficacious, even in participants with proliferative lupus nephritis and high levels of proteinuria. This is an important finding given the known correlations between glucocorticoid exposure, organ damage, and all-cause mortality.^28,32–35^

In addition to improved complete renal response rates, participants treated with voclosporin in this study also demonstrated significantly higher rates of partial renal response at 6 months (74% versus 47%, respectively, OR, 2.95; 95% CI, 1.41 to 6.19; *P* = 0.004). The significant difference between treatment arms in partial renal response was not maintained at 12 months (65% versus 51%, OR, 1.60; 95% CI, 0.80 to 3.20; *P* = 0.18). This may represent an additional level of heterogeneity in the population, which may require the inclusion of a larger, more targeted patient cohort in future studies to further characterize the therapeutic response over time in this subgroup.

In this study, we also investigated the time to achieve proteinuria reductions. The median time to achieve a ≥50% reduction in UPCR from baseline was within the first month of treatment for voclosporin-treated participants, consistent with the overall AURORA 1 population.^29^ However, additional time was required for participants with high levels of proteinuria to achieve UPCR ≤0.5 g/g relative to the overall AURORA 1 population. This is consistent with other studies in lupus nephritis that have demonstrated a correlation between higher levels of proteinuria and longer time to disease resolution.^14,36^ Yet, the addition of voclosporin to MMF and low-dose glucocorticoids still resulted in a significant benefit for participants with high levels of proteinuria.

The overall safety profile of voclosporin in participants with proliferative lupus nephritis and high levels of proteinuria was comparable with that of the control arm and the overall AURORA 1 study population, with no unexpected adverse events.^29^ Overall, most adverse events were mild to moderate in severity, and the number of serious adverse events reported was similar between the treatment arms.

Calcineurin inhibitors are known to cause hemodynamic changes resulting in intraglomerular vasoconstriction, and, as expected, the incidence of adverse events of GFR decreased was higher in participants on voclosporin relative to control; one participant in each arm had a serious adverse event of GFR decreased.^37,38^ The percentage of participants in each arm experiencing a laboratory-confirmed reduction in eGFR ≥30% from baseline was similar (voclosporin, 16% versus control, 18%). Furthermore, mean eGFR levels remained stable in both arms throughout the study period.

The overall rates of study drug discontinuation were higher in the control arm of this study (voclosporin, 29% versus control, 39%) and similar to those observed in the overall AURORA 1 study (voclosporin, 24% versus control, 33%).^29^

This study is not without limitations. Because this patient population may require a longer time on therapy to achieve a clinical response, the 12-month duration of the AURORA 1 study may not allow for a full assessment of the long-term efficacy and safety of voclosporin in this patient population. It is reassuring that a significant clinical benefit was observed in participants with UPCR ≥3 g/g treated with voclosporin even within the 12-month study period. Regarding longer-term voclosporin use, safety data are available from the AURORA 2 continuation study, which included 216 participants who continued randomized treatment with either voclosporin or placebo, in combination with MMF and low-dose glucocorticoids, for an additional 2 years after completion of the AURORA 1 study.^39^ An analysis of the overall population has revealed a similar safety profile in both study arms with 3 years of continued treatment, with the overall rate of adverse events decreasing over the study period.

While all participants included in this analysis were diagnosed with active lupus nephritis by kidney biopsy before study entry, activity and chronicity scores and pathology findings were not recorded for all participants. Although proteinuria remains one of the few available and most commonly used biomarkers of disease severity in lupus nephritis, it is not sufficient to fully characterize the duration and extent of disease in these patients. Studies have shown that proteinuria may not always correlate with renal histology, and patients with proteinuria <0.5 g/g may still have persistent lupus activity.^40–42^ Furthermore, the severity of lupus nephritis may have varied between the treatment arms despite similar baseline disease characteristics. However, given the strong correlation between nephrotic-range proteinuria and worse outcomes in lupus nephritis, examination of the safety and efficacy of voclosporin in patients with a UPCR of ≥3 g/g was warranted.

Finally, as a *post hoc* analysis of the AURORA 1 trial, this analysis was not powered to detect differences in treatment outcomes for this subset of patients. Despite this and lack of randomization specific to those with proliferative lupus nephritis and high levels of proteinuria, this analysis is informative and clinically relevant. It is also reassuring that when the analyses were applied to the AURORA 1 cohort with proteinuria <3 g/g, the results were similar with a smaller effect size. In addition, AURORA 1 was a global study that enrolled a diverse population of participants across races, ethnicities, and geographical regions, many of which have been associated with lower rates of renal response.^29,43–46^ Despite the study including many patient populations traditionally considered more difficult to treat, a significant benefit was reported with voclosporin in this substudy of patients with proliferative lupus nephritis and high levels of proteinuria, providing evidence of the benefit of voclosporin across the clinical spectrum.

Study participants with proliferative lupus nephritis and high levels of proteinuria treated with voclosporin in combination with MMF and low-dose glucocorticoids demonstrated significantly earlier reductions in proteinuria and higher rates of complete renal response compared with those treated with MMF and low-dose glucocorticoids alone over a 12-month period. Similar safety outcomes were reported in both the treatment and control arms. This analysis suggests that voclosporin is an effective therapy in this patient population.

## Supplementary Material

SUPPLEMENTARY MATERIAL

## Data Availability

Data can be shared upon request to corresponding author and signing data sharing agreement.

## Supplemental Material

Supplemental Table 1. Baseline demographics and disease characteristics in participants with UPCR <3 g/g.

Supplemental Table 2. Reasons for study drug discontinuation in participants with proliferative disease and UPCR ≥3 g/g.

Supplemental Table 3. Per-protocol glucocorticoid use and mean daily glucocorticoid dose in participants with proliferative disease and UPCR ≥3 g/g.

Supplemental Table 4. Complete renal response rates at 12 months controlling for baseline UPCR.

Supplemental Table 5. Immunology parameters at baseline and month 12 in participants with proliferative disease and UPCR ≥3 g/g.

Supplemental Table 6. Adverse events by preferred term leading to study drug discontinuation in participants with proliferative disease and UPCR ≥3 g/g.

Supplemental Table 7. Adverse events in participants with UPCR <3 g/g.

Supplemental Table 8. Adverse events by System Organ Class in participants with proliferative disease and UPCR ≥3 g/g.

Supplemental Figure 1. Study design of AURORA 1.

Supplemental Figure 2. Per-protocol oral glucocorticoid taper.

## References

[1] Hanly JG Su L Urowitz MB, . A longitudinal analysis of outcomes of lupus nephritis in an international inception cohort using a multistate model approach. Arthritis Rheumatol. 2016;68(8):1932–1944. doi:10.1002/art.39674 26991067 PMC5858760

[2] Tektonidou MG Dasgupta A Ward MM. Risk of end-stage renal disease in patients with lupus nephritis, 1971-2015: a systematic review and Bayesian meta-analysis. Arthritis Rheumatol. 2016;68(6):1432–1441. doi:10.1002/art.39594 26815601 PMC5071782

[3] Mahajan A Amelio J Gairy K, . Systemic lupus erythematosus, lupus nephritis and end-stage renal disease: a pragmatic review mapping disease severity and progression. Lupus. 2020;29(9):1011–1020. doi:10.1177/0961203320932219 32571142 PMC7425376

[4] Reich HN Gladman DD Urowitz MB, . Persistent proteinuria and dyslipidemia increase the risk of progressive chronic kidney disease in lupus erythematosus. Kidney Int. 2011;79(8):914–920. doi:10.1038/ki.2010.525 21248713

[5] Maroz N Segal MS. Lupus nephritis and end-stage kidney disease. Am J Med Sci. 2013;346(4):319–323. doi:10.1097/MAJ.0b013e31827f4ee3 23370533

[6] Lech M Anders H-J. The pathogenesis of lupus nephritis. J Am Soc Nephrol. 2013;24(9):1357–1366. doi:10.1681/ASN.2013010026 23929771 PMC3752952

[7] Fu SM Wang H Dai C Sung SSJ Gaskin F. Pathogenesis of proliferative lupus nephritis from a historical and personal perspective. Clin Immunol. 2017;185:51–58. doi:10.1016/j.clim.2016.07.024 27591148 PMC5332347

[8] Nolin AC Mulhern RM Panchenko MV, . Proteinuria causes dysfunctional autophagy in the proximal tubule. Am J Physiol Renal Physiol. 2016;311(6):F1271–F1279. doi:10.1152/ajprenal.00125.2016 27582098 PMC5210197

[9] Davies DJ Messina A Thumwood CM Ryan GB. Glomerular podocytic injury in protein overload proteinuria. Pathology. 1985;17(3):412–419. doi:10.3109/00313028509105494 2415904

[10] Cravedi P Remuzzi G. Pathophysiology of proteinuria and its value as an outcome measure in chronic kidney disease. Br J Clin Pharmacol. 2013;76(4):516–523. doi:10.1111/bcp.12104 23441592 PMC3791975

[11] Rovin BH Furie R Teng YKO, . A secondary analysis of the Belimumab International Study in Lupus Nephritis trial examined effects of belimumab on kidney outcomes and preservation of kidney function in patients with lupus nephritis. Kidney Int. 2022;101(2):403–413. doi:10.1016/j.kint.2021.08.027 34560137

[12] Liu T Neuner R Thompson A, . Clinical pharmacology considerations for the approval of belimumab for the treatment of adult patients with active lupus nephritis: a regulatory perspective. Lupus. 2022;31(4):424–432. doi:10.1177/09612033221079771 35238725

[13] McDonald S Yiu S Su L, . Predictors of treatment response in a lupus nephritis population: lessons from the Aspreva Lupus Management Study (ALMS) trial. Lupus Sci Med. 2022;9(1):e000584. doi:10.1136/lupus-2021-000584 35640982 PMC9157342

[14] Touma Z Urowitz MB Ibañez D Gladman DD. Time to recovery from proteinuria in patients with lupus nephritis receiving standard treatment. J Rheumatol. 2014;41(4):688–697. doi:10.3899/jrheum.130005 24429170

[15] Fanouriakis A Kostopoulou M Andersen J, . EULAR recommendations for the management of systemic lupus erythematosus: 2023 update. Ann Rheum Dis. 2023. doi:10.1136/ard-2023-224762. Epub ahead of print. PMID: 37827694 38580396

[16] Korbet SM Lewis EJ Schwartz MM Reichlin M Evans J Rohde RD. Factors predictive of outcome in severe lupus nephritis. Lupus Nephritis Collaborative Study Group. Am J Kidney Dis. 2000;35(5):904–914. doi:10.1016/s0272-6386(00)70262-9 10793026

[17] Turin TC James M Ravani P, . Proteinuria and rate of change in kidney function in a community-based population. J Am Soc Nephrol. 2013;24(10):1661–1667. doi:10.1681/ASN.2012111118 23833255 PMC3785273

[18] Qin X Hu H Cen J Wang X Wan Q Wei Z. Association between urinary protein-to-creatinine ratio and chronic kidney disease progression: a secondary analysis of a prospective cohort study. Front Med (Lausanne). 2022;9:854300. doi:10.3389/fmed.2022.854300 35433766 PMC9008575

[19] Gansevoort RT Matsushita K van der Velde M, . Lower estimated GFR and higher albuminuria are associated with adverse kidney outcomes. A collaborative meta-analysis of general and high-risk population cohorts. Kidney Int. 2011;80(1):93–104. doi:10.1038/ki.2010.531 21289597 PMC3959732

[20] Hemmelgarn BR Manns BJ Lloyd A, . Relation between kidney function, proteinuria, and adverse outcomes. JAMA. 2010;303(5):423–429. doi:10.1001/jama.2010.39 20124537

[21] Dall'Era M Stone D Levesque V Cisternas M Wofsy D. Identification of biomarkers that predict response to treatment of lupus nephritis with mycophenolate mofetil or pulse cyclophosphamide. Arthritis Care Res. 2011;63(3):351–357. doi:10.1002/acr.20397 21080348

[22] Dall'Era M Levesque V Solomons N Truman M Wofsy D. Identification of clinical and serological factors during induction treatment of lupus nephritis that are associated with renal outcome. Lupus Sci Med. 2015;2(1):e000089. doi:10.1136/lupus-2015-000089 26023331 PMC4442174

[23] Ugolini-Lopes MR Seguro LPC Castro MXF, . Early proteinuria response: a valid real-life situation predictor of long-term lupus renal outcome in an ethnically diverse group with severe biopsy-proven nephritis? Lupus Sci Med. 2017;4(1):e000213. doi:10.1136/lupus-2017-000213 29238603 PMC5724342

[24] Tamirou F Lauwerys BR Dall'Era M, . A proteinuria cut-off level of 0.7 g/day after 12 months of treatment best predicts long-term renal outcome in lupus nephritis: data from the MAINTAIN Nephritis Trial. Lupus Sci Med. 2015;2(1):e000123. doi:10.1136/lupus-2015-000123 26629352 PMC4654096

[25] Koo H Kim S Chin HJ. Remission of proteinuria indicates good prognosis in patients with diffuse proliferative lupus nephritis. Lupus. 2016;25(1):3–11. doi:10.1177/0961203315595130 26159540

[26] Kidney Disease: Improving Global Outcomes (KDIGO) Lupus Nephritis Work Group. KDIGO 2024 clinical practice guideline for the management of lupus nephritis. Kidney Int. 2024;105(1S):S1–S69. doi:10.1016/j.kint.2023.09.002 38182286

[27] van Gelder T Lerma E Engelke K Huizinga RB. Voclosporin: a novel calcineurin inhibitor for the treatment of lupus nephritis. Expert Rev Clin Pharmacol. 2022;15(5):515–529. doi:10.1080/17512433.2022.2092470 35763288

[28] Rovin BH Solomons N Pendergraft WF, . A randomized, controlled double-blind study comparing the efficacy and safety of dose-ranging voclosporin with placebo in achieving remission in patients with active lupus nephritis. Kidney Int. 2019;95(1):219–231. doi:10.1016/j.kint.2018.08.025 30420324

[29] Rovin BH Teng YKO Ginzler EM, . Efficacy and safety of voclosporin versus placebo for lupus nephritis (AURORA 1): a double-blind, randomised, multicentre, placebo-controlled, phase 3 trial. Lancet. 2021;397(10289):2070–2080. doi:10.1016/S0140-6736(21)00578-X 33971155

[30] Arriens C Teng YKO Ginzler EM, . Update on the efficacy and safety profile of voclosporin: an integrated analysis of clinical trials in lupus nephritis. Arthritis Care Res (Hoboken). 2023;75(7):1399–1408. doi:10.1002/acr.25007 36039949

[31] Hošková L Málek I Kopkan L Kautzner J. Pathophysiological mechanisms of calcineurin inhibitor-induced nephrotoxicity and arterial hypertension. Physiol Res. 2017;66(2):167–180. doi:10.33549/physiolres.933332 27982677

[32] Einarsdottir MJ Ekman P Molin M, . High mortality rate in oral glucocorticoid users: a population-based matched cohort study. Front Endocrinol (Lausanne). 2022;13:918356. doi:10.3389/fendo.2022.918356 35872995 PMC9304700

[33] Mejía-Vilet JM Ayoub I. The use of glucocorticoids in lupus nephritis: new pathways for an old drug. Front Med (Lausanne). 2021;8:622225. doi:10.3389/fmed.2021.622225 33665199 PMC7921306

[34] Zeher M Doria A Lan J, . Efficacy and safety of enteric-coated mycophenolate sodium in combination with two glucocorticoid regimens for the treatment of active lupus nephritis. Lupus. 2011;20(14):1484–1493. doi:10.1177/0961203311418269 21976398

[35] Furie R Rovin BH Houssiau F, . Two-year, randomized, controlled trial of belimumab in lupus nephritis. N Engl J Med. 2020;383(12):1117–1128. doi:10.1056/NEJMoa2001180 32937045

[36] Korbet SM Lewis EJ, Collaborative Study Group. Complete remission in severe lupus nephritis: assessing the rate of loss in proteinuria. Nephrol Dial Transplant. 2012;27(7):2813–2819. doi:10.1093/ndt/gfr741 22199359

[37] Barros EJ Boim MA Ajzen H Ramos OL Schor N. Glomerular hemodynamics and hormonal participation on cyclosporine nephrotoxicity. Kidney Int. 1987;32(1):19–25. doi:10.1038/ki.1987.166 3306096

[38] Bobadilla NA Tapia E Franco M, . Role of nitric oxide in renal hemodynamic abnormalities of cyclosporin nephrotoxicity. Kidney Int. 1994;46(3):773–779. doi:10.1038/ki.1994.332 7996799

[39] Saxena A Ginzler EM Gibson K, . Safety and efficacy of long‐term voclosporin treatment for lupus nephritis in the Phase 3 AURORA 2 clinical trial. Arthritis Rheumatol. 2024;76(1):59–67. doi:10.1002/art.42657 37466424 10.1002/art.42657

[40] Carlucci PM Li J Fava A, . High incidence of proliferative and membranous nephritis in SLE patients with low proteinuria in the Accelerating Medicines Partnership. Rheumatology. 2022;61(11):4335–4343. doi:10.1093/rheumatology/keac067 35212719 PMC9629353

[41] Weeding E Fava A Magder L Goldman D Petri M. One-third of patients with lupus nephritis classified as complete responders continue to accrue progressive renal damage despite resolution of proteinuria. Lupus Sci Med. 2022;9(1):e000684. doi:10.1136/lupus-2022-000684 35512816 PMC9047706

[42] De Rosa M Rocha AS De Rosa G Dubinsky D Almaani SJ Rovin BH. Low-grade proteinuria does not exclude significant kidney injury in lupus nephritis. Kidney Int Rep. 2020;5(7):1066–1068. doi:10.1016/j.ekir.2020.04.005 32647764 PMC7335958

[43] Isenberg D Appel GB Contreras G, . Influence of race/ethnicity on response to lupus nephritis treatment: the ALMS study. Rheumatology (Oxford). 2010;49(1):128–140. doi:10.1093/rheumatology/kep346 19933596 PMC2789586

[44] Contreras G Lenz O Pardo V, . Outcomes in African Americans and Hispanics with lupus nephritis. Kidney Int. 2006;69(10):1846–1851. doi:10.1038/sj.ki.5000243 16598205

[45] Barr RG Seliger S Appel GB, . Prognosis in proliferative lupus nephritis: the role of socio-economic status and race/ethnicity. Nephrol Dial Transplant. 2003;18(10):2039–2046. doi:10.1093/ndt/gfg345 13679478

[46] Adler M Chambers S Edwards C Neild G Isenberg D. An assessment of renal failure in an SLE cohort with special reference to ethnicity, over a 25-year period. Rheumatology (Oxford). 2006;45(9):1144–1147. doi:10.1093/rheumatology/kel039 16527882

