# Supplementary material for: Efficacy of Voclosporin in Proliferative Lupus Nephritis with High Levels of Proteinuria
Source: Clin J Am Soc Nephrol. 2023 Dec 18;19(3):309–18. doi: 10.2215/CJN.0000000000000297 (PMC10937024; doi:10.2215/CJN.0000000000000297)
Supplement: SUPPLEMENTARY MATERIAL [file cjasn-19-309-s001.pdf]

Efficacy of Voclosporin in Proliferative Lupus Nephritis with High Levels of Proteinuria - Supplemental Material

Table of Contents

Supplemental Tables *2*

Supplemental Table 1 Baseline Demographics and Disease Characteristics in Participants with UPCR <3 g/g 2

Supplemental Table 2 Reasons for Study Drug Discontinuation in Participants with Proliferative Disease and UPCR ≥3 g/g 3

Supplemental Table 3 Per-protocol Glucocorticoid Use and Mean Daily Glucocorticoid Dose in Participants with Proliferative Disease and UPCR ≥3 g/g 3

Supplemental Table 4 Complete Renal Response Rates at 12 Months Controlling for Baseline UPCR 4

Supplemental Table 5 Immunology Parameters at Baseline and Month 12 in Participants with Proliferative Disease and UPCR ≥3 g/g 5

Supplemental Table 6 Adverse Events by Preferred Term Leading to Study Drug Discontinuation in Participants with Proliferative Disease and UPCR ≥3 g/g 6

Supplemental Table 7 Adverse Events in Participants with UPCR <3 g/g 7

Supplemental Table 8 Adverse Events by System Organ Class in Participants with Proliferative Disease and UPCR ≥3 g/g 7

Supplemental Figures *8*

Supplemental Figure 1 Study Design of AURORA 1 8

Supplemental Figure 2 Per-protocol Oral Glucocorticoid Taper* 8

Supplemental Tables

Supplemental Table 1 Baseline Demographics and Disease Characteristics in Participants with UPCR <3 g/g

	UPCR <3 g/g	
	**Control**<br>**n=86**	**Voclosporin**<br>**n=84**
Age, mean (SD) years	35 (11)	34 (12)
Female sex, n (%)	71 (82.6)	78 (92.9)
Race, n (%)		
Asian[a]	25 (29.1)	27 (32.1)
Black	9 (10.5)	11 (13.1)
Other[b]	17 (19.8)	13 (15.5)
White	35 (40.7)	33 (39.3)
Ethnicity, n (%)		
Hispanic or Latino	27 (31.4)	25 (29.8)
Not Hispanic or Latino	58 (67.4)	59 (70.2)
Unknown	1 (1.2)	0
Time since lupus nephritis diagnosis, mean (SD) years	4.4 (5.4)	4.4 (5.9)
Biopsy Class, n (%)		
Class III	15 (17.4)	12 (14.3)
Class IV	37 (43.0)	41 (48.8)
Class III/V	11 (12.8)	9 (10.7)
Class IV/V	13 (15.1	10 (11.9)
Class V	10 (11.6)	11 (13.1)
Class III/IV	0	1 (1.2)
UPCR, mean (SD) g/g	2.1 (0.5)	2.0 (0.4)
eGFR, mean (SD) mL/min/1.73 m^2	89 (29)	98 (30)

Analysis includes participants from AURORA 1 (all biopsy classes) with baseline UPCR <3 g/g.
[a]Asian race includes Asian Indian, Chinese, Filipino, Japanese, Korean, Vietnamese, and Other Asian.
[b]Other race includes American Indian, Alaska Native, Native Hawaiian, Pacific Islander, and other or mixed races except mixed Black race.
eGFR, estimated glomerular filtration rate; SD, standard deviation; UPCR; urine protein creatinine ratio.

Supplemental Table 2 Reasons for Study Drug Discontinuation in Participants with Proliferative Disease and UPCR ≥3 g/g

	Control<br>n=72<br>n (%)	Voclosporin<br>n=76<br>n (%)
Completed study	59 (81.9)	68 (89.5)
Completed study on study drug	44 (61.1)	54 (71.1)
Discontinued study drug early	28 (38.9)	22 (28.9)
Adverse event	14 (19.4)	12 (15.8)
Death	1 (1.4)	0
Withdrew consent	3 (4.2)	3 (3.9)
Protocol non-compliance	0	2 (2.6)
Pregnancy	0	1 (1.3)
Physician decision	2 (2.8)	0
Prohibited medication required	0	1 (1.3)
Lack of efficacy	6 (8.3)	2 (2.6)
Other	2 (2.8)	1 (1.3)

Analysis includes patients from AURORA 1 with Class III or IV (± Class V lesions) lupus nephritis and baseline UPCR ≥3 g/g. Adverse events occurred on or after the day of the first dose and up to 30 days after the last dose of study drug, with the exception of death; includes all deaths post-randomization until completion of study follow-up.

Supplemental Table 3 Per-protocol Glucocorticoid Use and Mean Daily Glucocorticoid Dose in Participants with Proliferative Disease and UPCR ≥3 g/g

	Control<br>n=72	Voclosporin<br>n=76
Week 16, n (%)<br>≤2.5 mg/day	51 (79.7)	64 (86.5)
6 months, n (%)<br>≤2.5 mg/day	54 (85.7)	58 (86.6)
12 Months, n (%)<br>≤2.5 mg/day	33 (70.2)	35 (66.0)
Mean Daily Dose, mg<br>Mean (SD)<br>Median	8.8 (5.9)<br>5.2	8.1 (6.5)<br>5.2

Analysis includes participants from AURORA 1 with Class III or IV (± Class V lesions) lupus nephritis and baseline UPCR ≥3 g/g. Values are number (percentage) calculated out of number of participants in study at time point. Intravenous methylprednisolone 0.5 g/day was administered on Days 1 and 2 of both studies; oral glucocorticoid was initiated on Day 3 with 20-25 mg/day prednisone and adjusted per protocol-defined tapering schedule to a target dose of ≤2.5 mg/day at Week 16. SD, standard deviation.

Supplemental Table 4 Complete Renal Response Rates at 12 Months Controlling for Baseline UPCR

	Control	Voclosporin	Odds Ratio (95% CI) vs. Control	p-value
**UPCR ≥3 g/g (Class III/IV ± Class V)**				
	n=72	n=76		
CRR at 12 Months<br>Yes<br>No	 8 (11.1)<br>64 (88.9)	 26 (34.2)<br>50 (65.8)	4.16 (1.73, 9.97)	0.001
**UPCR <3 g/g (All Biopsy Classes)**				
	n=86	n=84		
CRR at 12 Months<br>Yes<br>No	 28 (32.6)<br>58 (67.4)	 41 (48.8)<br>43 (51.2)	1.98 (1.06, 3.68)	0.03
**UPCR <3 g/g (Class III/IV ± Class V)**				
	n=76	n=73		
CRR at 12 Months<br>Yes<br>No	 26 (34.2)<br>50 (65.8)	 34 (46.6)<br>39 (53.4)	1.68 (0.87, 3.24)	0.13
**UPCR <3 g/g (Class V)**				
	n=10	n=11		
CRR at 12 Months<br>Yes<br>No	 2 (20.0)<br>8 (80.0)	 7 (63.6)<br>4 (36.4)	7.0 (0.97, 50.57)	0.05

Analysis uses a logistic regression model with terms for treatment group, covariate, and treatment by covariate interaction. OR >1 demonstrates a treatment benefit of voclosporin. CI, confidence interval; CRR, complete renal response (defined aas UPCR ≤0.5 g/g with stable renal function [eGFR ≥60 mL/min/1.73 m^2 or no decrease >20% from baseline] in the presence of sustained, low-dose glucocorticoids [in the eight weeks prior to assessment] and no rescue medication) OR, odds ratio; UPCR, urine protein creatinine ratio.

Supplemental Table 5 Immunology Parameters at Baseline and Month 12 in Participants with Proliferative Disease and UPCR ≥3 g/g

	Control n=72		Voclosporin n=76	
	**Baseline**	**Month 12**	**Baseline**	**Month 12**
**Complement 3**				
n	72	58	76	67
Mean (SD) mg/dL	79.3 (35.8)	98.9 (33.8)	76.5 (32.4)	96.4 (32.1)
Low <90 mg/dL, n (%)	48 (66.7)	20 (27.8)	48 (63.2)	32 (42.1)
**Complement 4**				
n	72	58	76	67
Mean (SD) mg/dL	16.4 (10.6)	21.1 (13.8)	17.1 (9.0)	22.2 (10.9)
Low <10 mg/dL, n (%)	22 (30.6)	10 (13.9)	19 (25.0)	6 (7.9)
**Anti-dsDNA**				
n	72	58	75	65
Mean (SD) IU/mL	106.8 (133.5)	51.3 (64.1)	114.9 (125.8)	45.6 (57.8)
High ≥10 IU/dL, n (%)	49 (68.1)	36 (50.0)	64 (84.2)	53 (69.7)

Analysis includes patients from AURORA 1 with Class III or IV (± Class V lesions) lupus nephritis and baseline UPCR ≥3 g/g. Percentages calculated based on total number of patients with available data at each time point. Anti-dsDNA, anti-double-stranded DNA; SD, standard deviation.

Supplemental Table 6 Adverse Events by Preferred Term Leading to Study Drug Discontinuation in Participants with Proliferative Disease and UPCR ≥3 g/g

	Control n=72 n (%)	Voclosporin n=76 n (%)
Any adverse event leading to drug discontinuation	15 (20.8)[a]	11 (14.5)[b]
Renal impairment	2 (2.8)	3 (3.9)
Lupus nephritis	5 (6.9)	1 (1.3)
Renal failure	1 (1.4)	1 (1.3)
Glomerulonephritis	0	1 (1.3)
Chronic kidney disease	1 (1.4)	0
Proteinuria	1 (1.4)	0
GFR decreased	2 (2.8)	1 (1.3)
Electrocardiogram QT prolonged	0	1 (1.3)
Cervix carcinoma stage 0	0	1 (1.3)
Uterine leiomyoma	0	1 (1.3)
Pulmonary tuberculosis	0	1 (1.3)
Pneumonia	2 (2.8)	0
Urinary tract infection	1 (1.4)	0
Anemia	0	1 (1.3)
Hyperkalemia	0	1 (1.3)
Photosensitivity reaction	0	1 (1.3)
Hypertension	0	1 (1.3)
Pulmonary mass	1 (1.4)	0

[a]One patient had an adverse event (AE) of urinary tract infection leading to an action of 'Drug withdrawn'; the reason for study drug discontinuation was 'Physician Decision'. [b]One patient discontinued due to reason of 'Adverse Event' but did not have an AE leading to study drug withdrawal.

Analysis includes patients from AURORA 1 with Class III or IV (± Class V lesions) lupus nephritis and baseline UPCR ≥3 g/g. AEs occurred on or after the day of the first dose and up to 30 days after the last dose of study drug, with the exception of death; includes all deaths post-randomization until completion of study follow-up. AEs were aggregated by System Organ Class and Preferred Term and were coded using Medical Dictionary for Regulatory Activities v20.0.

GFR, glomerular filtration rate.

Supplemental Table 7 Adverse Events in Participants with UPCR <3 g/g

	Control<br>n=86<br>n (%)	Voclosporin<br>n=84<br>n (%)
Adverse Event (AE)	75 (87.2)	72 (85.7)
Serious Adverse Event (SAE)	18 (20.9)	14 (16.7)
Treatment-related SAE	4 (4.7)	4 (4.8)
AE leading to study drug discontinuation	10 (11.6)	7 (8.3)
AE leading to Death	2 (2.3)	0
Treatment-related AE leading to death	0	0

Analysis includes patients from AURORA 1 (all biopsy classes) and baseline UPCR <3 g/g. Adverse events defined as an adverse event that occurs on or after the day of the first dose and up to 30 days after the last dose of study drug, with the exception of death; includes all deaths post-randomization until completion of study follow-up.

Supplemental Table 8 Adverse Events by System Organ Class in Participants with Proliferative Disease and UPCR ≥3 g/g

	Control<br>n=72<br>n (%)	Voclosporin<br>n=76<br>n (%)
**AEs by System Organ Class and reported in ≥5% of patients in either treatment arm[a]**		
Infections and infestations	42 (58.3)	47 (61.8)
Gastrointestinal disorders	29 (40.3)	38 (50.0)
Investigations	10 (13.9)	31 (40.8)
Nervous system disorders	11 (15.3)	17 (22.4)
Renal and urinary disorders	21 (29.2)	14 (18.4)
Blood and lymphatic system disorders	12 (16.7)	13 (17.1)
Metabolism and nutrition disorders	20 (27.8)	13 (17.1)
General disorders and administration site conditions	14 (19.4)	12 (15.8)
Musculoskeletal and connective tissue disorders	15 (20.8)	12 (15.8)
Vascular disorders	11 (15.3)	12 (15.8)
Respiratory, thoracic, and mediastinal disorders	10 (13.9)	11 (14.5)
Skin and subcutaneous tissue disorders	16 (22.2)	10 (13.2)

[a]Patients are counted only once per System Organ Class.
Analysis includes patients from AURORA 1 with Class III or IV (± Class V lesions) lupus nephritis and baseline UPCR ≥3 g/g. Adverse events occurred on or after the day of the first dose and up to 30 days after the last dose of study drug, with the exception of death; includes all deaths post-randomization until completion of study follow-up. AEs were aggregated by System Organ Class and Preferred Term and were coded using Medical Dictionary for Regulatory Activities v20.0.
AE, adverse event.

Supplemental Figures

Supplemental Figure 1 Study Design of AURORA 1

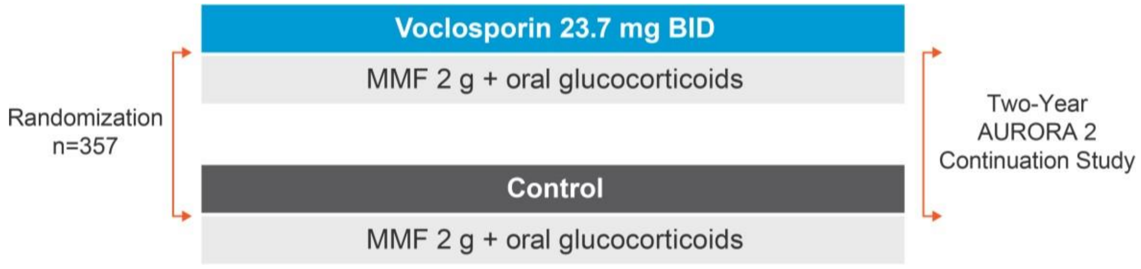

The current study includes patients from AURORA 1 with Class III or IV (± Class V lesions) lupus nephritis and baseline UPCR ≥3 g/g. Of the original AURORA 1 population (n=357), 76 patients in the voclosporin arm and 72 patients in the control arm were included in the current study. BID, twice daily; MMF, mycophenolate mofetil; UPCR, urine protein creatinine ratio.

Supplemental Figure 2 Per-protocol Oral Glucocorticoid Taper*

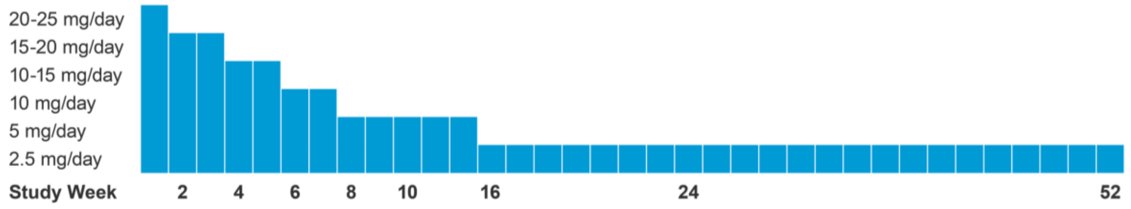

*Intravenous methylprednisolone 0.5 g/day administered on Days 1 and 2. Oral glucocorticoid initiated on Day 3 with 20-25 mg/day prednisone and rapidly tapered to a target dose of 2.5 mg/day at Week 16.
